# Solanaceous Crops-Derived Nitrogen-Doped Biomass Carbon Material as Anode for Lithium-Ion Battery

**DOI:** 10.3390/nano15171357

**Published:** 2025-09-03

**Authors:** Hong Shang, Yougui Zhou, Huipeng Li, Jia Peng, Xinmeng Hao, Lihua Guo, Bing Sun

**Affiliations:** School of Science, China University of Geosciences (Beijing), Beijing 100083, China; zyg2119220051@163.com (Y.Z.); lhp155487@163.com (H.L.); p19803395505@163.com (J.P.); hxm206121@163.com (X.H.); huahua990403@163.com (L.G.)

**Keywords:** biomass-derived carbon, nitrogen doping, anode material, lithium-ion battery

## Abstract

Biomass resources are excellent candidates for carbon electrode materials due to their abundance, renewability, and biodegradability. Herein, the solanaceous crop *Tobacco Straw*, a rich agricultural by-product, was utilized to prepare biomass-derived carbon material (TsC) and applied as an anode in lithium-ion batteries (LIBs). Doping or composite formation is considered to enhance the electrochemical performance. Doping extra nitrogen (N) atoms into the TsC (denoted as TsNC) demonstrated exceptional reversible specific capacity (475.9 mA h g^−1^ at the current density of 60 mA g^−1^ after 500 cycles) and remarkable long-term cycling stability (142.9 mA h g^−1^ even at a high current density of 1.5 A g^−1^ after 1000 cycles, much larger than that of TsC), attributed to the increased lithium-ion (Li-ion) adsorption sites including graphitic-N, pyrrolic-N, and pyridinic-N. Furthermore, kinetic analysis revealed that a prominent predominant surface capacitive-controlled behavior was responsible for the superior rate performance of TsNC, which could facilitate rapid charging and discharging at high rates. This work offers valuable insights into the application and modification of nitrogen-doped biomass-derived carbons with outstanding electrochemical properties for LIBs. The strategy also sheds light on enabling waste recycling and generating economic benefits.

## 1. Introduction

Sustainable energy storage devices, such as supercapacitors [1], fuel cells [2], and batteries [3], have received widespread attention and achieved significant advancements to face the challenges of environmental and energetic issues [4]. Among them, LIBs have been successfully applied in various aspects of human life due to their low pollution, long cycle life, and high energy density [5]. In LIBs, electrode materials are crucial components. Currently, graphite carbon is the most widely used commercial material for the anode [6]. Exploring abundant and renewable carbon materials for LIB anodes is desirable for developing more low-cost, high-performance, sustainable energy storage devices.

Biomass resources are environmentally friendly, abundant, and renewable, making them a great choice for preparing low-cost carbon materials [7]. Utilizing biomass-derived materials promotes waste recycling and generates favorable economic benefits [8]. Biomass-derived carbon materials, consisting of lignin, hemicellulose, and cellulose, feature unique porous, hierarchical structures and rich heteroatom-active sites, benefiting them as appropriate anode materials in LIBs [9]. Recently, biomass-derived carbons are serving as anode materials by proposing various strategies, including doping, composite formation, and activation, to enhance electrochemical performance. For example, carbon materials derived from polyvinylidene fluoride (PVDF)-coated cellulose by being pyrolyzed at 800 °C demonstrate the highest specific capacity of approximately 200 mA h g^−1^ at a current density of 2000 mA g^−1^ [10]. Hydrothermal carbonization can be employed to prepare cross-linked carbon nanospheres, which raises their specific surface areas and provides favorable conditions for subsequent doping [11]. Furthermore, chemical activation [12], including the use of alkalis (KOH), acids (H_3_PO_4_) [13], alkali metal carbonates (K_2_CO_3_) [14], and metal salts (CaCl_2_) [15], is widely applied to enhance porosity, defects, and specific surface area [16]. The generation of new micropores and mesopores can be achieved through the chemical activators above, where the amount of activator controls the pore structure and distribution in the carbon material, thus affecting Li-ion adsorption and storage.

More importantly, biomass-derived carbons have the advantage of introducing heteroatoms (such as N [17,18,19], P [20,21], and O [22,23]), which enhances the specific capacity of the battery by supplying more adsorption active sites. Notably, nitrogen doping has become one of the most promising strategies for enhancing LIB’s performance due to the enhanced electrical conductivity of the material, additional structural defects, and redox-active sites, providing abundant Li-ion adsorption capacity and thereby increasing the energy density of LIBs [24]. Theoretical calculations indicate that the binding energies of pyrrole-N and pyridine-N to Li atoms are 4.46 eV and 4.26 eV, respectively, which are significantly higher than graphite carbon (3.64 eV) [25]. The stronger binding energy facilitates better Li-ion adsorption ability for enhancing electrochemical performance [26]. Currently, various external nitrogen source additions or self-doping methods have been developed to develop modified biomass-derived carbon for LIB anodes [27,28]. For example, immersing graphite flakes in urea leads to a porous structure with rich heteroatoms, reaching a capacity of 806.6 mA h g^−1^ at 30 mA g^−1^ [29]. Similarly, nitrogen-doped porous carbon with an extremely high nitrogen content of 18.79% was synthesized through hydrothermal treatment and subsequent post-treatment with a nitrogen source, exhibiting excellent rate performance and cycling stability [30]. These advancements highlight the effectiveness of subsequent nitrogen doping in optimizing the performance of carbon-based anode materials.

**Scheme 1:**
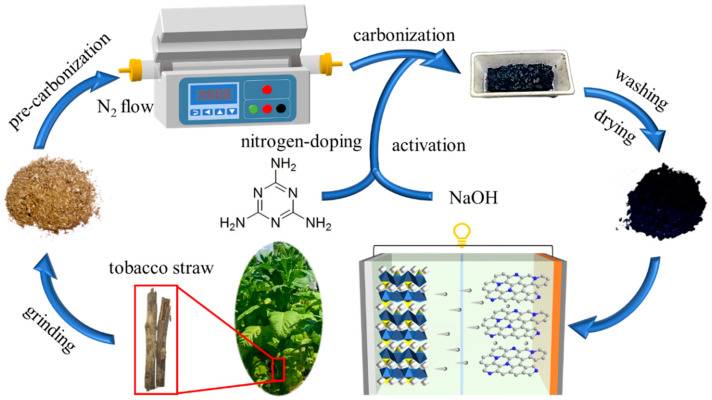
Schematic illustration of the preparation of TsNC.

*Tobacco Straw* is a by-product of the manufacturing of cigarettes and contains abundant pristine nitrogen elements. Herein, nitrogen-enriched biomass-derived carbons from *Tobacco Straw* (TsC and TsNC) were successfully prepared (Scheme 1), which exhibit excellent electrochemical properties as anode materials for LIB. It was applied as the raw biomass material due to its natural nitrogen content, enabling it to display a reasonable battery capacity even without nitrogen doping. The addition of melamine followed by carbonization enhanced the nitrogen content, which significantly improved the electrochemical performance in LIB. Owing to its amorphous carbon nanostructure, abundant microporous and mesoporous structures, and numerous defects, TsNC demonstrated excellent first discharge specific capacity (723.4 mA h g^−1^ at 60 mA g^−1^) and cycling stability (142.9 mA h g^−1^ at 1.5 A g^−1^ after 1000 cycles). Especially, there is still a capacity of 475.9 mA h g^−1^ remaining at a current density of 60 mA g^−1^ after 500 cycles, without any attenuation. This work emphasizes the promise of nitrogen-rich biomass for Li storage and underscores the impact of modification in boosting the efficiency of energy storage devices.

## 2. Experimental

### 2.1. Preparation of TsC and TsNC

Initially, the *Tobacco Straw* was cut into pieces and washed with deionized water. After cleaning, the straw was completely dried in an oven at 100 °C for 12 h and then crushed into fine powder. The powder was heated to 500 °C in a tubular furnace with a nitrogen atmosphere for 2 h with a heating rate of 5 °C/min in order to carbonize the lignin. The chemical activation and nitrogen doping processes were carried out simultaneously. After the powder had cooled to room temperature, it was mixed with sodium hydroxide and melamine powder in a 1:1:1 ratio and then subjected to a grinding process in an agate mortar for 5 min. Subsequently, the mixture was heated for a second time in a tubular furnace under conditions of nitrogen atmosphere, reaching 600 °C for 1 h with a heating rate of 5 °C/min. Finally, the resulting TsNC product was washed several times with diluted hydrochloric acid and deionized water to neutralize the pH and remove remaining impurities, followed by drying at 100 °C for 12 h. The preparation process for *Tobacco Straw* carbon (TsC) was nearly identical to that for TsNC, except for the nitrogen doping process.

### 2.2. Materials Characterization

Based on nitrogen adsorption–desorption isotherms, the Brunauer–Emmett–Teller (BET) method was utilized to calculate the specific surface area and pore size distribution. Hitachi Model S-4800 field emission scanning electron microscope (SEM) fitted with an energy-dispersive spectrometer (EDS) was applied to analyze the surface morphology and elemental distribution (Hitachi High-Tech Corporation, Tokyo, Japan). Transmission electron microscope (TEM) and high-resolution transmission electron microscope (HRTEM) images were captured with an HT-7700 electron microscope at an accelerating voltage of 100 kV (Ibaraki, Japan). The structure was analyzed using X-ray diffraction (XRD) patterns obtained from an Empyrean diffractometer with Cu Kα radiation (λ = 1.5406 Å) at 45 kV and 40 mA (Malvern Panalytical, Malvern, UK). Raman spectroscopy, utilizing a Renishaw-2000 Raman spectrometer and a 473 nm Ar laser, was employed to access the defects in carbon materials (Wotton-under-Edge, UK). To determine the element composition of the carbon materials, X-ray photoelectron spectroscopy (XPS) was performed with the Thermo Scientific ESCALAB 250Xi apparatus (Boston, MA, USA).

### 2.3. Electrochemical Measurement

The activated carbon-based anodes were prepared by mixing activated carbon, carbon super P, and PVDF in a ratio of 90:5:5, respectively. The electrode materials were then coated onto the copper foil using N-methyl pyrrolidone (NMP) as the solvent. The working electrodes were dried at 60 °C for 6 h. CR2032 button cells were assembled in an Ar-filled glove box, using the electrode sheets coated with material, polypropylene membrane separators (Celgard 2400) (Celgard, Charlotte, NC, USA), Li foils, and 1.0 M LiPF_6_ electrolyte solution, prepared by dissolving ethylene carbonate (EC) and diethyl carbonate (DEC) in a 1:1 volume ratio. Cyclic voltammograms (CV) were recorded on a CHI760E electrochemical workstation between 0.01 and 3.0 V at various scan rates (0.5, 1.0, 2.0, 3.0, 4.0, and 5.0 mV s^−1^). Galvanostatic charge–discharge tests were performed within the potential ranging from 0.05 V to 3.0 V vs. Li/Li^+^ utilizing the CT2001A land battery test system. Electrochemical impedance spectroscopy (EIS) was measured over a frequency range from 100 kHz to 10 mHz.

## 3. Results and Discussion

### 3.1. Morphology and Structure Characterization

**Figure 1:**
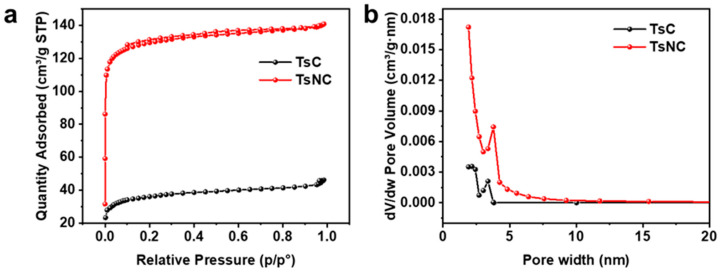
(**a**) Nitrogen adsorption–desorption isotherm and (**b**) pore size distribution of TsC and TsNC calculated from the adsorption isotherms using the BET method.

The Brunauer–Emmett–Teller (BET) surface area and porous nature of TsC and TsNC were characterized using the N_2_ adsorption–desorption method. As shown in Figure 1a, the isothermal curves for both carbon materials displayed a steep increase in nitrogen adsorption at low pressures, indicating a typical type *I* isotherm with an H_4_-type hysteresis loop. This behavior suggested the predominant presence of micropores in both TsC and TsNC. According to the data presented in Table S1, the specific surface areas were determined to be 114.28 m^2^ g^−1^ for TsC and 378.46 m^2^ g^−1^ for TsNC, with corresponding pore volumes of 0.071 cm^3^ g^−1^ and 0.216 cm^3^ g^−1^, respectively. Figure 1b showed the Barrett-Joyner-Halenda (BJH) pore size distributions for the two materials, revealing average pore sizes of 2.765 nm for TsC and 3.378 nm for TsNC. The large surface areas and the internal mesopore/micropore structures in TsC and TsNC carbon materials can accelerate the transportation/diffusion of Li-ion and mitigate the volume change in LIBs when used as electrode materials and contribute to perfect electrochemical performance.

**Figure 2:**
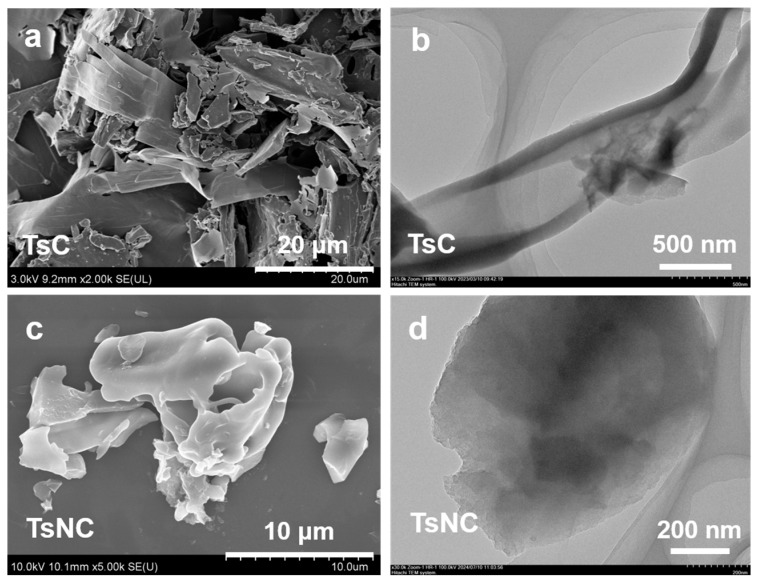
(**a**,**c**) SEM images of TsC and TsNC; (**b**,**d**) TEM images of TsC and TsNC.

The microscopic morphologies of the carbon materials TsC and TsNC were analyzed using scanning electron microscope (SEM), as shown in Figure 2a,c. Both materials exhibited fractured carbon block structures with irregularly stacked carbon layers. During the pyrolysis process, TsC fragmented into carbon blocks with uneven edges and smooth surfaces. Notably, the application of NaOH did not result in a substantial etching of the carbon surfaces. TsNC retained the fundamental structure of TsC, but its surfaces were marked by numerous bumps and holes [31]. These uneven surfaces resulted from the reaction between melamine and the biomass waste during the nitrogen doping process, further enhanced by NaOH etching. This morphology increases the specific surface area of the carbon black material, thereby exposing the internal pore structure, which is advantageous for the diffusion of Li-ion [32]. Transmission electron microscopy (TEM) was employed to reveal the structural characteristics. The tightly stacked carbon layers in TsC resulted in a structure with considerable thickness and an opaque interior, as shown in Figure 2b. In contrast, TsNC exhibited a similar carbon layer structure but with more irregular edges (Figure 2d). Furthermore, an energy-dispersive spectrometer (EDS) was conducted on the TsC and TsNC to demonstrate the presence and distribution of C, N, and O. All heteroatoms were found to be evenly dispersed within the carbon framework of both TsC and TsNC in Figures S1 and S2. Moreover, the remaining oxygen in the biomass-derived carbon materials was likely due to incomplete thermal decomposition during the preparation [33].

**Figure 3:**
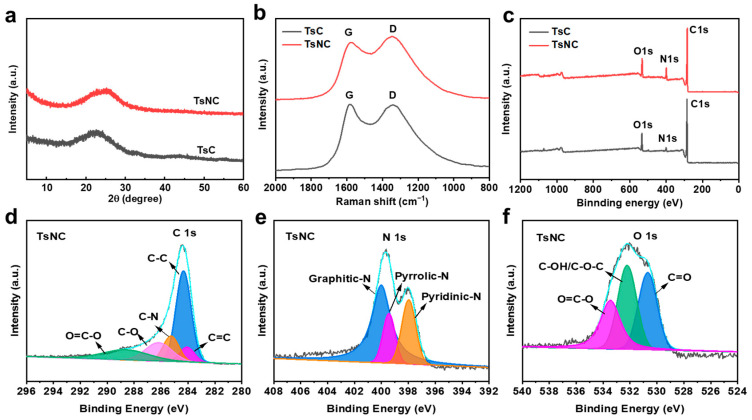
(**a**) XRD patterns of TsC and TsNC; (**b**) Raman spectra of TsC and TsNC; (**c**) XPS spectra of TsC and TsNC; (**d**–**f**) High-resolution spectra of C 1s, N 1s, and O 1s of TsNC.

To further investigate the impact of the nitrogen doping process on the structural characteristics, X-ray diffraction (XRD) patterns were conducted on the carbon materials. Figure 3a presented the XRD patterns of TsC and TsNC. Both of them showed weak diffraction peaks around 24°, suggesting that the carbon layers after carbonization are amorphous. The graphite (002) peak of the carbon appeared at 22.6° and shifted to 26.2° after nitrogen doping. Amorphous carbon can introduce defects such as edges and pores, which provide abundant active sites for the intercalation of Li-ion [34]. The layer spacing (*d*_002_) of TsC and TsNC was determined to be 0.393 nm and 0.340 nm, respectively, according to the Bragg Equation. In comparison with the layer spacing of graphite (*d*_002_ = 0.335 nm), both biomass-derived carbon materials exhibited larger layer spacing, which facilitated Li-ion transmission and enhanced Li-ion transfer efficiency [35]. Raman spectroscopy (Figure 3b) was used to analyze the graphitization degree and defects in TsC and TsNC. Two strong peaks were observed at 1342 cm^−1^ and 1583 cm^−1^, corresponding to the D and G peaks, respectively [36]. The D peak represented defects in the carbon lattice, while the G peak corresponds to the stretching vibration of sp^2^ C–C bonds. The degree of defects in carbon materials is often quantified by the intensity ratio of the D and G peaks (I_D_/I_G_) [37]. The I_D_/I_G_ ratios for TsC and TsNC were 0.992 and 1.098, respectively, indicating that TsNC has more defects due to the nitrogen doping and activation process. Structural features of carbon materials, such as edges, defects, and micropores, offer enhanced active sites for Li-ion storage [38]. To determine the composition of the carbons, X-ray photoelectron spectroscopy (XPS) was performed. The full XPS survey spectrum was shown in Figure 3c. Both carbon materials displayed strong peaks at 284.5 eV, 400.0 eV, and 532.5 eV, corresponding to the characteristic peaks of C 1s, N 1s, and O 1s, respectively. The content of nitrogen in TsNC was 8.13%, significantly higher than that of 3.37% in TsC. Figures S3–S5 and Figure 3d–f showed the high-resolution spectra of C 1s, N 1s, and O 1s of TsC and TsNC. The C 1s spectrum of TsNC in Figure 3d can be deconvoluted into five peaks at 283.61, 284.33, 285.25, 286.18, and 288.60 eV, corresponding to C=C, C–C, C–N, C–O, C=O, and O=C–O, respectively (Table S2). The nitrogen species can be classified into three binding states, including graphitic-N, pyrrolic-N, and pyridinic-N, at 400.01, 399.44, and 397.97 eV, respectively [39]. After nitrogen doping, the content of pyrrolic-N remained the highest among the nitrogen species (Table S3), and there was a significant increase in the proportion of graphitic-N and pyridinic-N in TsNC. The content of pyridinic-N in TsC (16.54%) was lower than TsNC (24.12%). The presence of pyridinic-N and pyrrolic-N contributes active sites for Li-ion storage, and pyridinic-N is more favorable than pyrrolic-N [40]. Meanwhile, the increase in graphitic-N enhanced the conductivity of TsNC. Thus, when used as electrode material, TsNC is expected to exhibit enhanced electrochemical performance.

### 3.2. Electrochemical Performance

**Figure 4:**
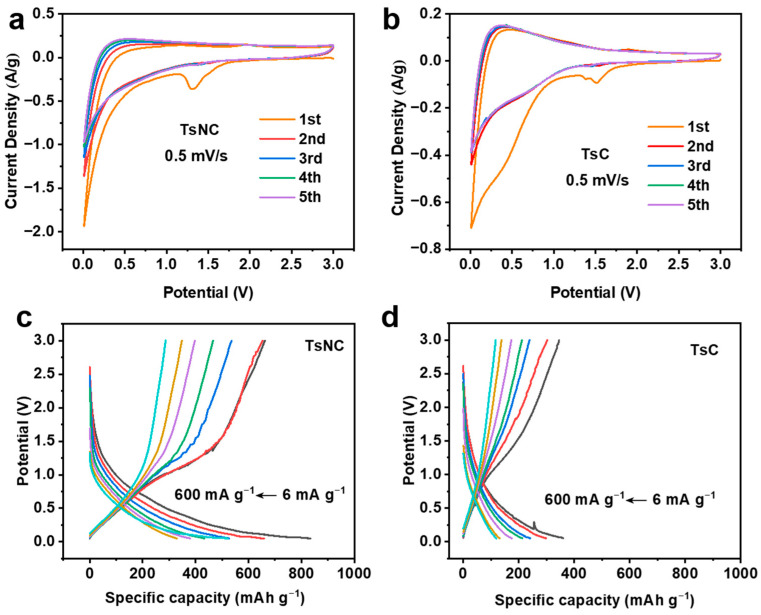
(**a**) Cyclic voltammetry curves of TsNC electrode at a scan rate of 0.5 mV·s^−1^; (**b**) Cyclic voltammetry curves of TsC electrode at a scan rate of 0.5 mV·s^−1^; (**c**,**d**) Galvanostatic charge–discharge curves of TsNC and TsC electrodes from 6 mA g^−1^ to 600 mA g^−1^.

The prepared TsC and TsNC were used as the anode materials to investigate the electrochemical performance. The cyclic voltammograms (CV) for the initial five cycles were shown in Figure 4a,b, with a scan rate of 0.5 mV s^−1^ and a voltage range of 0.01–3.0 V vs. Li/Li-ion. In the initial discharge cycle, the reduction peak for TsC was clearly observed at 1.5 V, while for TsNC, it shifted to a lower voltage of 1.3 V, corresponding to the adsorption of Li-ion by heteroatoms and defects [11]. This suggests that the addition of melamine alters the structure of the TsC. Irreversible current responses were observed in the range of 0–0.5 V, which are indicative of the formation of the solid electrolyte interface (SEI) and the decomposition of the electrolyte in LIBs. During the charge cycle, the oxidation peak around 0.3 V corresponded to the reversible deintercalation of Li-ion from the graphite layer, with the reaction Li^+^ + C↔LiC_6_. The CV curves after the first cycle overlapped significantly, indicating excellent cycle stability during subsequent charging and discharging. The galvanostatic charge–discharge profiles at current densities ranging from 6 mA g^−1^ to 600 mA g^−1^ over a voltage range of 0.01 to 3.0 V were shown in Figure 4c,d. The sloping curves demonstrate the capacitive behavior of both TsC and TsNC. Additionally, the two platforms near 1.3 V in the discharge curve were consistent with the CV measurement.

**Figure 5:**
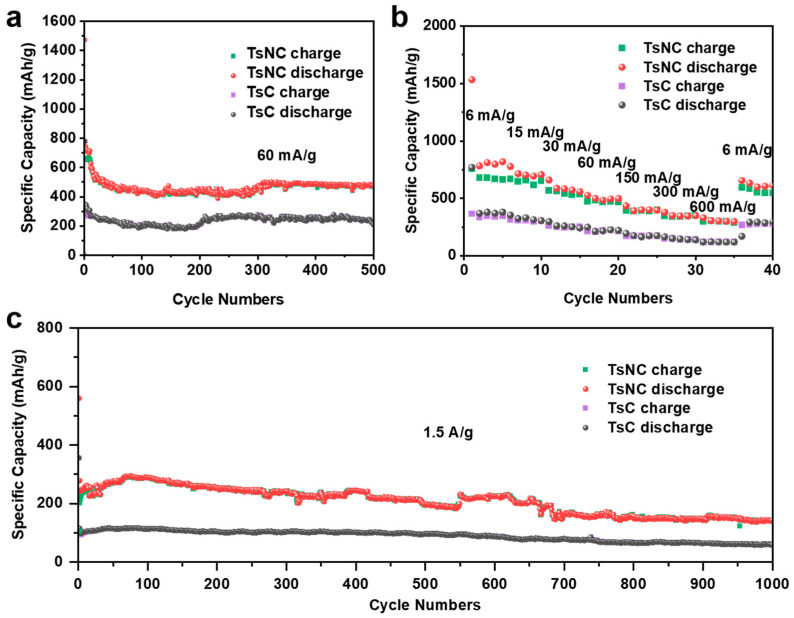
(**a**) Cycling performance of TsC and of TsNC electrodes at a current density of 60 mA g^−1^; (**b**) Comparison of the rate performance of TsC and TsNC; (**c**) Cycling performance of TsC and of TsNC electrodes at a current density of 1.5 A g^−1^.

The cycle stability of TsC and TsNC was further evaluated at 60 mA g^−1^ over the voltage range of 0.05–3.0 V, as shown in Figure 5a. TsC exhibited an initial discharge capacity of 780.2 mA h g^−1^ and a charge capacity of 346.4 mA h g^−1^, resulting in a coulombic efficiency of 44.4% for the first cycle. The low coulombic efficiency can be attributed to the formation of the SEI layer and irreversible Li loss during Li-ion insertion into the carbon lattice [41]. Similarly, TsNC demonstrated a first discharge capacity of 1472.5 mA h g^−1^ and a first charge capacity of 723.4 mA h g^−1^, accompanied by an initial coulombic efficiency of 49.1%. The elevated initial charge–discharge capacity and coulombic efficiency underscore the exceptional Li storage capacity and cycling stability of TsNC. The reversible specific capacity of TsNC remained at 475.9 mA h g^−1^ after 500 cycles at the current density of 60 mA g^−1^, which was significantly higher than the capacity of the TsC electrode (212.4 mA h g^−1^). Figure 5b showed the rate performance of the TsC and TsNC electrodes at various current densities. The values of the reversible capacities of TsNC at current densities of 6, 15, 30, 60, 150, 300, and 600 mA g^−1^ were 768.5, 706.2, 558.2, 498.4, 397.1, 349.3, and 298.0 mA h g^−1^, respectively. When the current density was returned to 6 mA g^−1^, the reversible capacity of TsNC quickly recovered to 611.7 mA h g^−1^, demonstrating its excellent Li-ion storage ability and rate performance [42]. In contrast, the TsC electrode displayed poor rate performance, with reversible capacities of 380.6, 305.5, 241.6, 221.4, 174.7, 137.4, and 119.1 mA h g^−1^ at the same current densities. Furthermore, the cycling stability was also evaluated at 1.5 A g^−1^, as shown in Figure 5c. TsNC maintained a high reversible capacity of 142.9 mA h g^−1^ after 1000 cycles, demonstrating its excellent cycle life and potential as an anode electrode for LIBs [43]. In comparison, TsC showed a much lower specific capacity of 59.9 mA h g^−1^ after 1000 cycles.

Electrochemical impedance spectroscopy (EIS) was conducted to assess the charge transfer and ion diffusion resistance of the TsC and TsNC electrodes (Figures S6 and S7). The semi-circular shape observed in the high-frequency region is indicative of the charge transfer resistance (*R*_ct_). In contrast, the line in the low-frequency region corresponds to the Warburg impedance (*Z*_w_), which is associated with Li-ion diffusion [44]. A smaller diameter of the half-circle in the high-frequency region indicates lower resistance. As shown in Figures S6 and S7, the *R*_ct_ of the TsNC electrode was lower than that of the TsC electrode. This improvement can be attributed to the stable SEI film formed during the charging and discharging processes, which effectively reduced interfacial impedance and preserved the stability of the porous structure. As a result, the TsNC electrode exhibited superior electrical conductivity compared to the TsC electrode.

Furthermore, CV was performed on TsC and TsNC anodes at different scan rates ranging from 0.5 to 5.0 mV s^−1^ to investigate the kinetic behavior and electrochemical reaction mechanism in LIBs (Figures S8 and S6). As the scanning rate increased, both the peak current and peak potential underwent a shift. All CV curves of TsNC displayed similar shapes, indicating that the intercalation and deintercalation behavior of Li-ion in TsNC is quasi-reversible [45]. Typically, the relationship between the peak current and scan rate follows Equation (1).

(1) $$i=av^{b}$$

(2) $$\log\left(i\right)=blog(v)+log(a)$$

**Figure 6:**
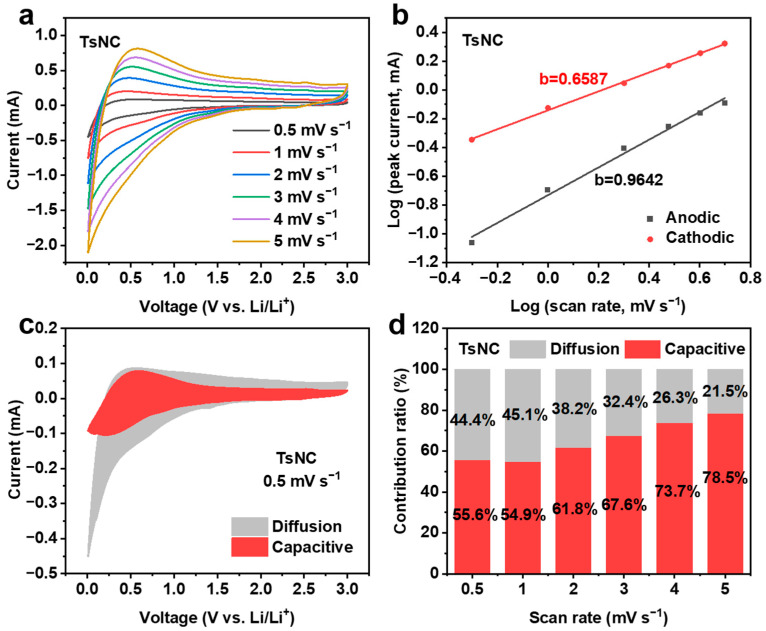
Kinetic analysis of the TsNC anode in LIBs. (**a**) CV curves at various scan rates from 0.5 to 5 mV s^−1^; (**b**) The measurement of b-value; (**c**) Contribution of the capacitive and diffusion process at a scan rate of 0.5 mV s^−1^; (**d**) Contribution ratios of the capacitive process at various scan rates.

Here, *a* and *b* represent unknown parameters, and the current is directly influenced by the scan rate. The value of parameter *b* can be determined from the linear relationship between log (*v*) and log (*i*) (Equation (2)). The slope *b* is indicative of the type of electrochemical behavior that governs the process. Generally, a *b*-value of 1 indicates a capacitive-controlled process, while a *b*-value of 0.5 suggests a diffusion-controlled process [46]. As shown in Figure 6b, the *b*-values were 0.9642 and 0.6587 for TsNC in the cathodic and anodic processes, respectively. This result indicated that the intercalation/deintercalation process of Li-ion in TsNC was hybrid-controlled by both capacitive and battery-type behavior [47]. Similarly, the *b*-values for TsC cathodic and anodic processes were 0.6096 and 0.8005, respectively, indicating that the Li storage mechanism of the electrode involves a mixed process of diffusion and capacitance (Figure S8). Notably, the *b*-value of the TsNC cathodic peak indicated a strong capacitive-type behavior, which can facilitate rapid charging and discharging at high rates. To further demonstrate the excellent rate performance of TsNC electrodes, the contributions of surface capacitance and ion diffusion were quantified by calculating the corresponding current in a specific voltage range, as described by Equation (3).

(3) $$i=k_{1}v+k_{2}v^{\frac{1}{2}}$$

(4) $$i/v^{\frac{1}{2}}=k_{1}v^{\frac{1}{2}}+k_{2}$$

in which *k*_1_*v* represents the contribution of capacitive behavior, and *k_2_v*^1/2^ represents the contribution of diffusion behavior [48,49]. The values *k*_1_ and *k*_2_ are obtained from cyclic voltammetry data of different scan rates. The capacitive contribution of the TsNC electrode was determined to be 55.6% at a scan rate of 0.5 mV s^−1^ (shown in the red area of Figure 6c). As the scanning rate frequency increased from 0.5 to 5.0 mV s^−1^, the capacitive contribution ratio of TsNC was 55.6%, 54.9%, 61.8%, 67.6%, 73.7%, and 78.5%, respectively (Figure 6d). In comparison, the maximum capacitance contribution of TsC at 5.0 mV s^−1^ was 57.6%. These results further indicated that the Li storage mechanism in TsNC is primarily governed by a capacitive-controlled process [50]. The high percentage of capacitive behavior in TsNC can be attributed to its amorphous carbon structure, abundant defects, and high pyridine-N content, all of which can promote Li-ion adsorption and contribute to its superior rate performance.

## 4. Conclusions

In summary, TsC and TsNC biomass waste-derived carbon materials were successful achieved through the utilization of solanaceous crops—*Tobacco Straw* as raw material and melamine as a nitrogen source—employing a pre-carbonization and pyrolysis process. The carbon materials exhibit porous structures, high nitrogen content, and abundant defects. After extra nitrogen doping, TsNC carbon occupied additional active sites, which enhanced the intercalation and storage of Li-ion. As anodes in LIBs, TsNC demonstrated excellent rate performance, cycling stability, and remarkable capacity, achieving 475.9 mA h g^−1^ at 60 mA g^−1^ even after 500 cycles. In contrast, TsC with natural nitrogen demonstrated a capacity of only 212.4 mA h g^−1^, demonstrating the important role of subsequent nitrogen doping. Furthermore, kinetic analysis revealed that lithium storage in TsNC is primarily governed by a capacitive process, underscoring its potential for rapid charging and discharging at high rates (142.9 mA h g^−1^ even at a high current density of 1.5 A g^−1^ after 1000 cycles, much larger than that of TsC). This work presents a cost-effective strategy for the recycling of biomass waste and highlights its potential applications in energy storage.

## Data Availability

The datasets used and/or analyzed during the current study are available from the corresponding author upon reasonable request.

## Supplementary Materials

The following supporting information can be downloaded at https://www.mdpi.com/article/10.3390/nano15171357/s1: Figure S1: SEM image of TsC and the corresponding elemental mapping images of C, N, and O; Figure S2: SEM image of TsNC and the corresponding elemental mapping images of C, N, and O; Figure S3: High-resolution C 1s spectra of TsC; Figure S4: High-resolution N 1s spectra of TsC; Figure S5: High-resolution O 1s spectra of TsC; Figure S6: Comparison of impedance spectra of TsC and TsNC before the cycle; Figure S7: Comparison of impedance spectra of TsC and TsNC after 500 cycles; Figure S8: (a) CV curves of TsC at various scan rates from 0.5 to 5 mV s^−1^; (b) The measurement of b-value of TsC; (c) Contribution of the capacitive and diffusion process of TsC at a scan rate of 0.5 mV s^−1^; (d) Contribution ratios of the capacitive process of TsC at various scan rates; Table S1: Porosity parameters of the TsC and TsNC materials; Table S2: XPS C1s analysis of the elemental composition of TsNC and TsC; Table S3: N1s elemental analysis of the TsC and TsNC materials; Table S4: Performance comparison of biochar carbon as anodes in LIBs. References [14,20,29,51] have been cited in the Supplementary Materials.
