# Supplementary material for: Solanaceous Crops-Derived Nitrogen-Doped Biomass Carbon Material as Anode for Lithium-Ion Battery
Source: Nanomaterials (Basel). 2025 Sep 3;15(17):1357. doi: 10.3390/nano15171357 (PMC12430359; doi:10.3390/nano15171357)
Supplement: Supplementary file 1 [file nanomaterials-15-01357-s001.zip › nanomaterials-3841332-supplementary-send conversion.pdf]

nanomaterials

MDPI

Solanaceous Crops-Derived Nitrogen-Doped Biomass Carbon Material as Anode for Lithium-Ion Battery

Hong Shang *, Yougui Zhou, Huipeng Li, Jia Peng, Xinmeng Hao, Lihua Guo and Bing Sun *

School of Science, China University of Geosciences (Beijing), Beijing 100083, China; zyg2119220051@163.com (Y.Z.); lhp155487@163.com (H.L.); p19803395505@163.com (J.P.); hxm206121@163.com (X.H.); huahua990403@163.com (L.G.)

* Correspondence: shanghong@cugb.edu.cn (H.S.); sunbing@cugb.edu.cn (B.S.)

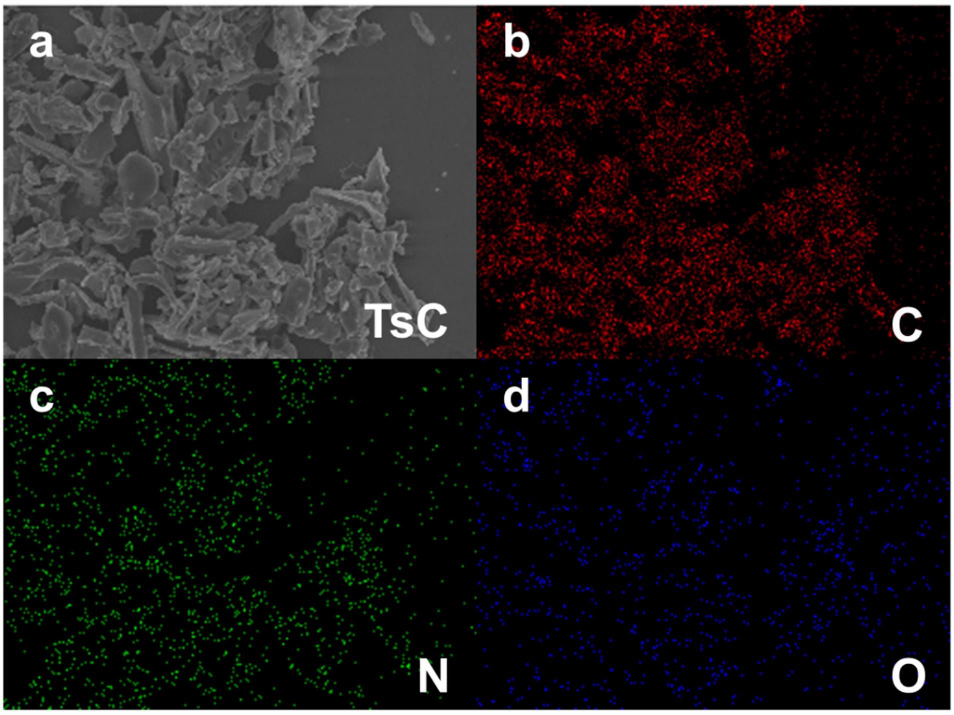

Figure S1. SEM image of TsC and the corresponding elemental mapping images of C, N, O.

 https://doi.org/10.3390/nano15171357

nanomaterials

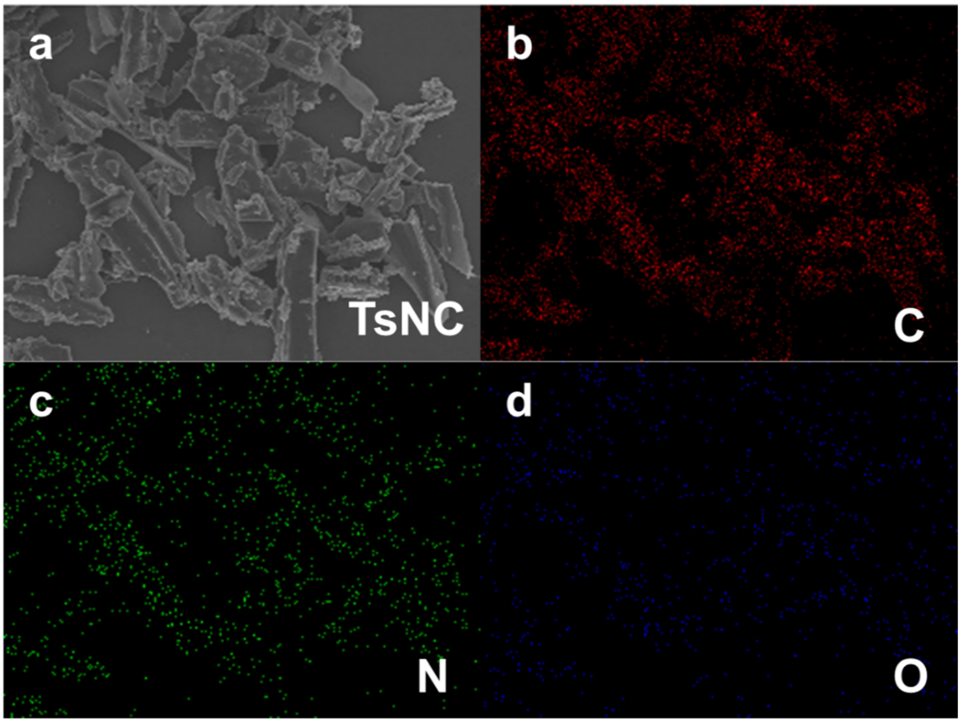

Figure S2. SEM image of TsNC and the corresponding elemental mapping images of C, N, O.

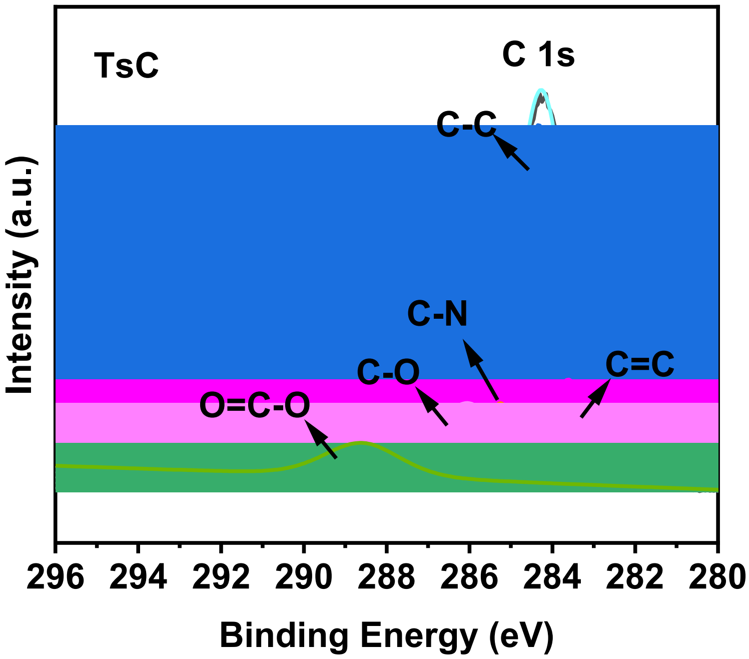

Figure S3. High-resolution C 1s spectra of TsC.

https://doi.org/10.3390/nano15171357

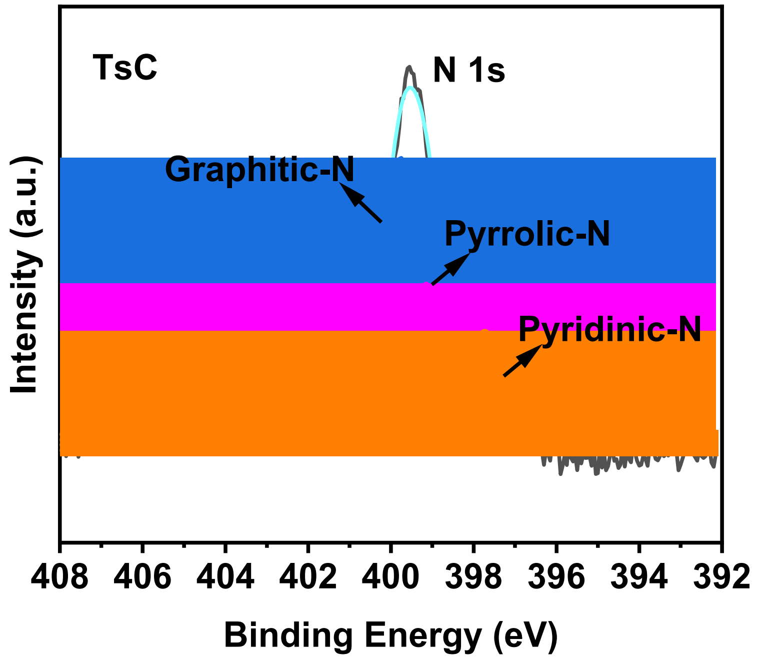

Figure S4. High-resolution N 1s spectra of TsC.

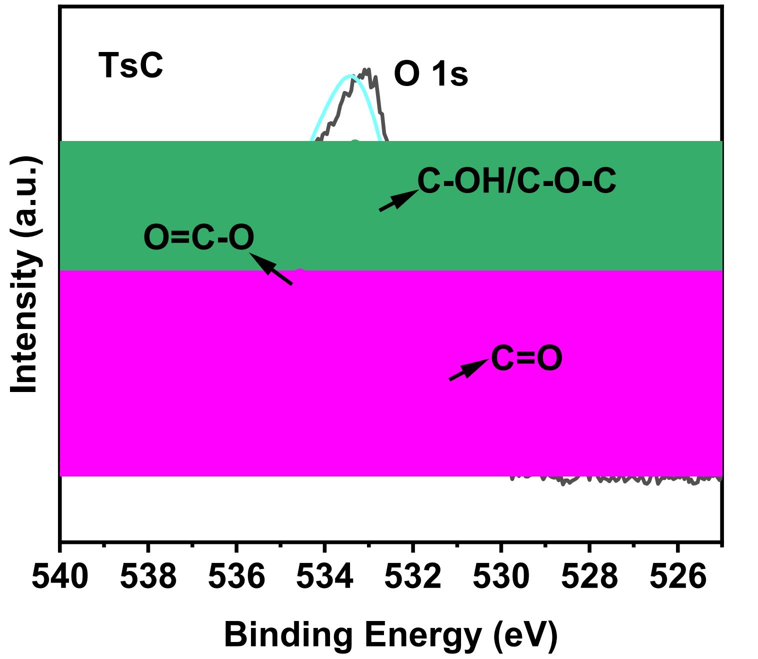

Figure S5. High-resolution O 1s spectra of TsC.

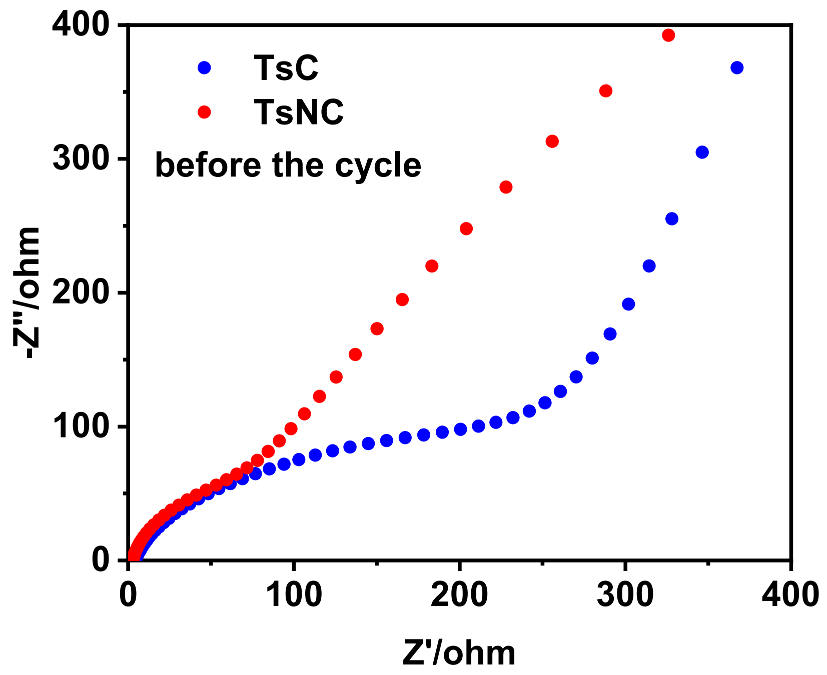

Figure S6. Comparison of impedance spectra of TsC and TsNC before the cycle.

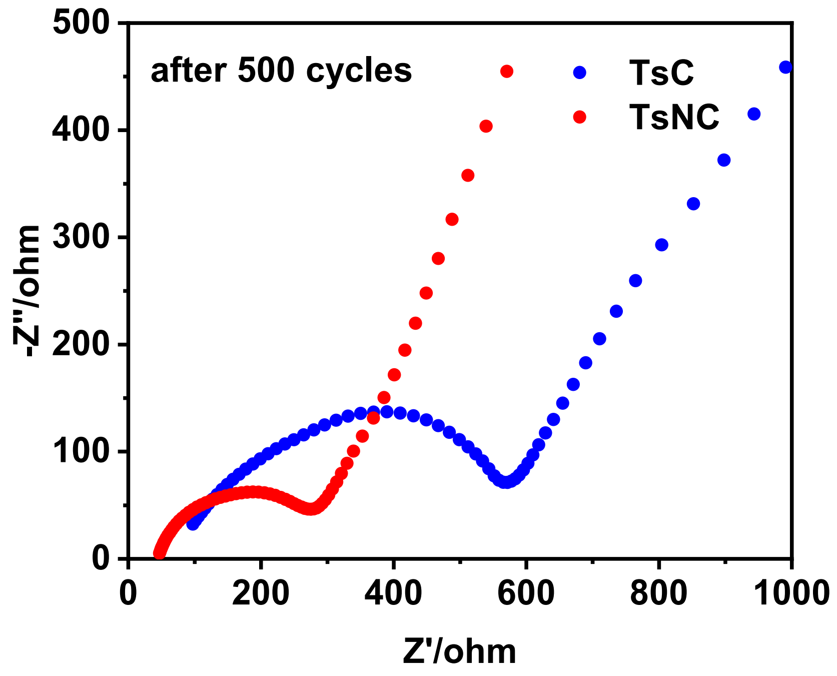

Figure S7. Comparison of impedance spectra of TsC and TsNC after 500 cycles.

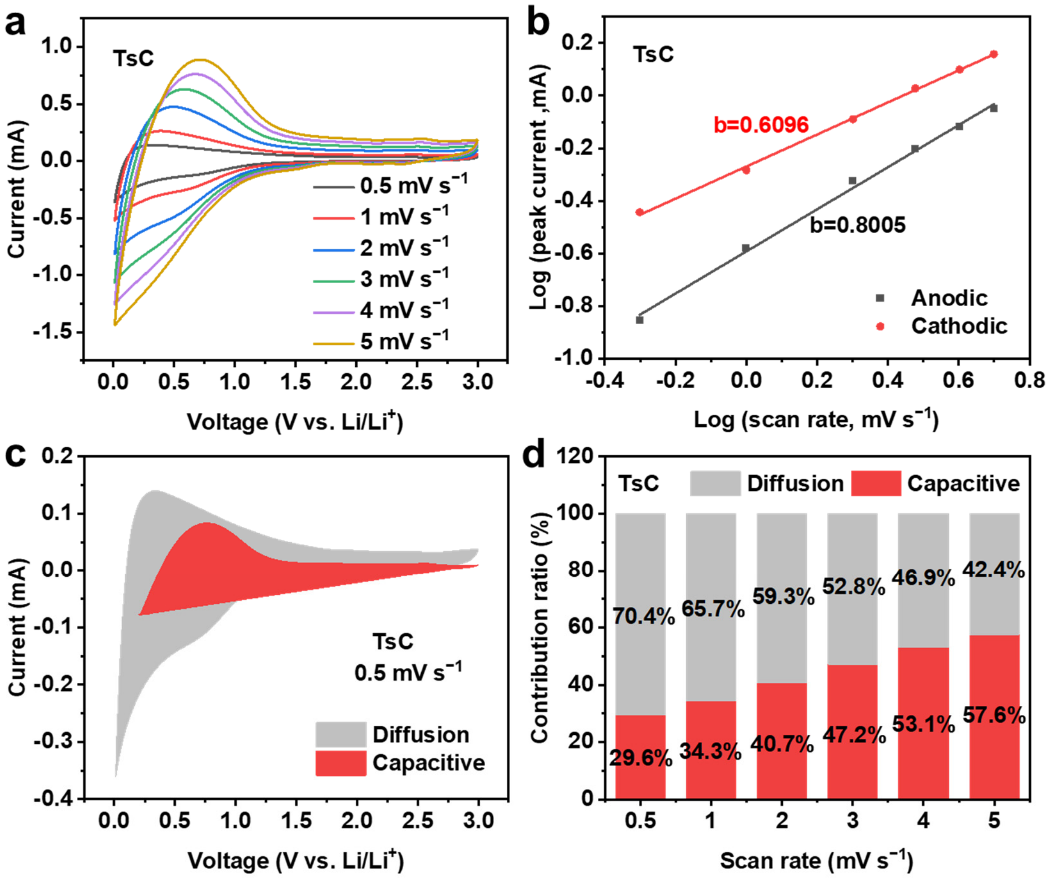

Figure S8. (a) CV curves of TsC at various scan rates from 0.5 to 5 mV s^{-1}; (b) The measurement of b-value of TsC; (c) Contribution of the capacitive and diffusion process of TsC at a scan rate of 0.5 mV s^{-1}; (d) Contribution ratios of the capacitive process of TsC at various scan rates.

Table S1. Porosity parameters of the TsC and TsNC materials.

Material	Specific surface area (m^2 g^{-1})	Total pore volume (cm^3 g^{-1})	Average pore size (nm)
TsC	114.2823	0.070854	2.7650
TsNC	378.4657	0.215631	3.3777

Table S2. XPS C1s analysis of the elemental composition of TsNC and TsC.

Material	Peaks and their positions (eV)				
	C=C	C-C	C-N	C-O	O=C-O
	283.61	284.33	285.25	286.18	288.60
TsC	12.54 %	52.37 %	8.26 %	16.55 %	10.28 %
TsNC	7.34 %	45.72 %	13.34 %	16.33 %	17.27 %

Table S3. N1s elemental analysis of the TsC and TsNC materials.

Material	N (atom%)	Graphitic-N	Pyrrolic-N	Pyridinic-N
TsC	3.37 %	64.10 %	19.37 %	16.54 %
TsNC	8.13 %	59.53 %	16.35 %	24.12 %

Table S4. Performance comparison of biochar carbon as anodes in LIBs.

Biomass	Functionality doping	Current density	Specific capacity	Ref.

TsC	natural N	60 mA g^{-1}<br>1.5 A g^{-1}	212.4 mA h g^{-1} after 500 cycles<br>59.9 mA h g^{-1} after 1000 cycles	this work
TsNC	extra N doping	60 mA g^{-1}<br>1.5 A g^{-1}	475.9 mA h g^{-1} after 500 cycles<br>142.9 mA h g^{-1} after 1000 cycles	this work
Spartina alterniflora	N	6 mA g^{-1}	412.9 mA h g^{-1} after 600 cycles	Nanomaterials, 2025, 15, 658
Hemp	N	30 mA g^{-1}	676.1 mA h g^{-1} after 45 cycles	Chem. Asian J. 2023, 18, e202300279
Pinecone	pristine	100 mA g^{-1}	306.6 mA h g^{-1} after 500 cycles	J. Power Sources 2023, 580, 233329
Lotus root	N, P	0.5 A g^{-1}	599.16 mA h g^{-1} after 215 cycles	Int. J. Hydrogen Energy 2024, 56, 828–836
